# Factors influencing adverse events reporting within the health care system: the case of artemisinin-based combination treatments in northern Ghana

**DOI:** 10.1186/s12936-016-1172-2

**Published:** 2016-02-27

**Authors:** Samuel Chatio, Raymond Aborigo, Philip Baba Adongo, Thomas Anyorigiya, Philip Ayizem Dalinjong, Patricia Akweongo, Abraham Oduro

**Affiliations:** Navrongo Health Research Centre, P.O Box 114, Navrongo, Ghana; School of Public Health, College of Health Sciences, University of Ghana, Legon, Ghana

**Keywords:** Adverse events reporting, Artemisinin-based combination therapy, Northern Ghana

## Abstract

**Background:**

The use of artemisinin-based combination therapy (ACT) as first-line treatment for uncomplicated malaria was a policy recommended by World Health Organization. In 2004, Ghana changed her first-line anti-malarial drug policy to use ACT. This study examined factors affecting adverse events reporting in northern Ghana after the introduction of ACT.

**Methods:**

This was a qualitative study based on sixty in-depth interviews with health workers, chemical shop owners and patients with malaria who were given ACT at the health facilities. Purposive sampling method was used to select study participants. The interviews were transcribed, coded into themes using Nvivo 9 software. The thematic analysis framework was used to analyse the data.

**Results:**

Study respondents reported body weakness and dizziness as the most frequent side effects they had experienced from the used of ACT. Other side effects they reported were swollen testes, abdominal pain and shivering. These side effects were mostly associated with the use of artesunate-amodiaquine compared to other artemisinin-based combinations. Patients were not provided information about the side effects of the drugs and so did not report when they experienced them. Also long queues at health facilities and unfriendly health worker attitude were the main factors affecting adverse events reporting. Other factors such as wrong use of ACT at home, farming and commercial activities also affected effective adverse events reporting in the study area.

**Conclusion:**

Patients’ lack of knowledge and health sector drawbacks affected side effect reporting on ACT. Intensive health education on likely side effects of ACT should be provided to patients by health workers. Also, improving health worker attitude toward clients will encourage patients to visit the health facilities when they react negatively to ACT and, subsequently, will improve on adverse events reporting.

## Background

The World Health Organization (WHO) in 2001 recommended to all malaria endemic countries to shift to artemisinin-based combination therapy (ACT) for the management of uncomplicated falciparum malaria [1]. Due to the urgency of the policy change, most health systems embraced the new policy without a proper understanding of the appropriateness of the different formulations of ACT for their contexts. The choice of an ACT was based primarily on available literature with limited trials in-country to determine their efficacy, cost effectiveness and the capacity of local industry to produce generic drugs [2, 3]. Consequently, research studies that evaluated the performance of the new anti-malarials reported widespread severe side effects such as severe headache, body weakness, dizziness and vomiting [4, 5]. Patients who experienced side effects were required to report to the nearest health facility, but compliance was low due to poor adverse events reporting systems [4]. An adverse event is any undesirable experience associated with the use of a medical product in a patient [6]. Patients with mild side effects were able to contain them at home and severe cases reported to health facilities. Although most of the causes of the side effects were drug-related, some were due to the introduction of generics that were poorly formulated and poor compliance to drug regimens [4].

Ghana changed her first-line anti-malarial drug policy in 2004, from chloroquine to the use of artesunate-amodiaquine as the first-line drug for the treatment of uncomplicated malaria [7]. The implementation process was faced with several challenges related to adverse events and lack of other treatment options [7]. Artemether-lumefantrine and dihydroartemisinin-piperaquine were subsequently added as first-line anti-malarial treatment for uncomplicated malaria to cater for those who could not tolerate the artesunate-amodiaqiune [7].

These different artemisinin-based combinations have been used to treat uncomplicated malaria in Ghana for about a decade now. However, surveillance on adverse events has not been encouraging. Data on adverse events of ACT has not been systematically collected and, therefore, little evidence exists in determining the effectiveness of the drugs in real life situations. This study was designed to explore the factors that influence adverse events reporting in two rural districts in northern Ghana. Specifically, the study obtained information on the adverse events of ACT, examined how the information is obtained from patients and makes recommendations for the setting up of a platform for reporting adverse events, especially for anti-malarials.

## Methods

### Study site

This study was conducted in the Kassena Nankana east and west Districts. The study area covers about 1675 square kilometres of Sahelian savannah with a population of about 153,000 under continuous demographic surveillance [8]. The area is predominantly rural with subsistence farming as the mainstay for the people. Malaria burden is seasonal with the high transmission period occurring between June–October coinciding with the rainy season [9, 10].

The health system in the area comprises one hospital, eight health centres/clinics, two private clinics and 28 Community-based Health Planning and Services (CHPS) compounds located in the communities and providing limited curative and preventive services [11, 12]. The district hospital located in Navrongo central serves as a referral hospital for all the health facilities operating in the two districts. There is one pharmacy shop and over 50 drug and chemical shops located in the study districts.

### Sampling of health facilities

The study area is divided into five zones (North, South, East, West and Central) based on their geographical locations by the NHDSS [8]. Based on these demarcations, two zones were randomly selected for the study. This was done by writing the names of the five zones on a piece of paper each and concealed by wrapping the paper. Two people randomly selected the first two zones which were north and south. The two health centres located in the two selected zones were used for the recruitment of study participants. The Government of Ghana launched the National health Insurance Scheme (NHIS) in 2004 to replace user fees or what is popularly known as “cash and carry” [13]. The health insurance scheme has ten accredited chemical shops in the study area. The only two accredited chemical shops located in the selected zones were also used as recruitment centres. In addition, the district hospital and the only pharmacy shop located in Navrongo town were also selected as recruitment sites for study participants due to their high patronage for malaria treatment in the districts.

### Sampling of respondents

All malaria patients who visited the study health facilities and received ACT during the period of data collection in the selected health facilities were recruited into the study. The data collectors spent 2 days in each of the selected health facilities/chemical shops and all patients with malaria who received ACT were identified and followed-up for interview at home. All qualified patients consented to be part of the study. The longest duration for taking ACT is 3 days. All recruited participants were therefore followed-up on the 4th day when they were expected to have completed their doses. Health workers/chemical shop owners in the study health facilities/chemical shops in the two zones were also selected for the in-depth interviews. The health professionals were selected to share their experiences on the side effects of ACT and the factors affecting side effects reporting.

### Data collection

The in-depth interviews were conducted by trained university graduates with experience in qualitative research technique. Addresses were obtained from study participants who were recruited at the study facilities to enable the data collectors visit them at home and conduct the interviews. Appointments were booked with all the study participants before the interviews were conducted. A total of sixty in-depth interviews were conducted in this study. Twenty interviews with health professionals and forty interviews with patients with malaria who were given ACT at the study facilities. The interviews were tape recorded with consent from participants. The interviews were conducted in Kasem, Nankani and English depending on the language the participant preferred.

### Data processing and analysis

The recorded interviews were transcribed verbatim and entered into a computer using Microsoft word. A coding list was prepared based on the objectives of the study. The transcripts were read and edited to make sure that all themes were covered during data collection. The data analysis was initiated simultaneously with data collection. This was to ensure that new emerging themes were incorporated into the guide and that thematic saturation was monitored. The data was organized using QSR Nvivo 9 software before analysis. A codebook was developed based on the major themes of the study. Quotes from the interviews were used to support themes discussed in the results.

### Ethical consideration

Ethical approval for the study was received from the Navrongo Health Research Centre Institutional Review Board. The approval number is NHRCIRB 152. In line with the approved procedure of obtaining consent for the study, oral consent was obtained from each participant prior to being interviewed and this was approved as part of the protocol for this study. Oral informed consent was obtained because the study processes posed minimal risk to study participants. This method of consent was solicited and obtained as the majority of the respondents had no formal education and those with formal education also opted for this method of consent. The verbal consent was voice recorded with the agreement of participants preceding to each interview. The data collectors read and translated the consent form into the preferred local language of each participant on the purpose of the study, study procedure, right to withdraw and efforts to ensure confidentiality. The oral consenting process was recorded on a separate voice recorder from the one used for the actual interview. In addition, they were made to recommend a member of the household to witness the consenting process and the demographic data of the witnesses were collected. Participants were also told that the findings would be published in a peer review journal. All children from 10 to 17 years old gave assent while their parents/caretakers gave consent before the interviews were conducted.

## Results

### Experience of side-effects of ACT

The findings revealed that participants who took artesunate-amodiaqiune to treat malaria reported most side effects. The most commonly reported side effects were general body weakness and dizziness. Participants associated poor eating before taking their medications as the main cause of the side effects. Others however thought that the components of artesunate-amodiaqiune were too high for them and, therefore, the side effects. Some patients shared their experiences in the excerpts below.

*“I was given artesunate*-*amodiaquine and when I started taking the drug I could not do anything because my whole body was weak”* (IDI: 29-year old male patient).

The other side effects reported were vomiting, chest pains, itching, loss of appetite, poor vision and shivering. Some participants said they experienced an unusual heart beat and their health deteriorated after taking artesunate-amodiaquine.

*“….When I took the artesunate*-*amodiaquine, I had chest pains and I vomited*” (IDI: 32-year old female patient).

Other uncommon side effects of ACT were also reported by one of the participants. As she put it:

*“.…others especially the men have their testes and body swollen. We got this experience from our father who died of malaria” (*IDI: 32-year old female patient).

Similar views were shared by health professionals and chemical shop owners on the side effects patients with malaria experience from using ACT. A 38-year old health worker had this to say on the issue:

*“Well, some patients vomit and there is dizziness as well, but it is the dizziness and the vomiting that I have heard a lot of people complained about. Some people complain of poor vision and difficulty to hear or they experience some noise in their ears”*(IDI: 38-year old health worker).

The health workers explained that some malaria patients only initiate treatment when the condition is severe and that this was responsible for some of the side effects and not necessarily ACT. They were particularly concerned about overdosing of medications, including ACT, by some patients as a contributor to these side effects.

*“The other reason is that they think that when they take overdose it will help to cure the illness fast and this makes them get the side effect. Those who are insured, when they collect these drugs they go and share them with those who are not insured at home and such people will not be able to educate them the way they will take the drug hence they can use the drug the way they want and when they get these side effects, they turn to put the blame on the drug*” (IDI: 32-year old health worker).

Table 1 contains summary of reported side effects of artesunate-amodiaquine by study participants.

**Table 1:** Reported side effects of Artesunate-Amodiaquine

Side effect	Quote
Body weakness	*“I was given artesunate-amodiaquine and when I started taking the drug I could not do anything because my whole body was weak”* (IDI-29 year old male patient)
Vomiting and dizziness	*“Well, some patients vomit and there is dizziness as well, but it is the dizziness and the vomiting that I have heard a lot of people complained about. Some people complain of poor vision and difficulty to hear or they experience some noise in their ears”* (IDI-38 year old health worker)
Chest pains	*“…When I took the artesunate-amodiaquine, I had chest pains and I vomited”* (IDI-32 year old female patient)
Swollen testicles	*“…others especially the men have their testes and body swollen. We got this experience from our father who died of malaria. When it starts unless he takes akpetashie (type of wine prepared locally) (type of wine prepared locally) it will always be serious”* (IDI-32 year old female patient)
Loss of appetite	*“When my child took the artesunate-amodiaquine, he was weak and could not open his eyes. For me, immediately I put it into my mouth, I become very weak… when I took it the first time, I lost appetite and I was also very weak”* (IDI-26 year old Mother).
Shivering and poor vision	*“Some people complain of poor vision and difficulty to hear or they hear some noise in their ears”* (IDI-36 year old health worker)

### Consultation content during out-patient visits

The consultation content between clinician and client in the Out-Patient Department (OPD) focused mainly on drug administration to the neglect of drug efficacy and side effects. Only a few patients reported receiving information on side effects of the ACT that was given but none reported being told to report back to the health facility if they experienced any side effects. They said that providers emphasized on the drug regimen and occasionally shared information on prevention methods for malaria.

*“They did not tell me anything apart from the way I should use the medicine. This is my second time of going there (health facility) and they have not told me anything on the side effects I will get when I use the drug”***(**IDI: 25-year old female patient).

The providers confirmed that in addition to giving instructions on the dosing regimen, they are required to provide information on side effects to patients and to explain to them why it was important for them to report any side effects they experience with the drug to the nearest health facility. They however said that they rarely share such information with their clients due to the high patient numbers that characterize the OPDs daily. This was clearly conveyed by one of the health workers in the following quote:

*“Yes, as for that one, it is true, some don’t have the time to do the education and I think it is Out Patient Department (OPD) congestion that is causing that. Sometimes the OPDs will be too full with patients seeking for health care services that you think if I should waste time doing education, I cannot finish early. I think that is the reason because all of us have been trained on the counseling but we are not doing because of the pressure on us”* (IDI: 26-year old health worker).

### Adverse events reporting system

According to the health workers, reporting of adverse drug events within the health system is spontaneous. Health workers solicit the events from their clients with the expectation that the clients will comply. All reported events are captured using a specially designed form from the Ghana Food and Drugs Authority (FDA). Pharmacists at the various health facilities have the primary responsibility of collating all reported events, which are then forwarded to the FDA office within the region for advice. The FDA is required to examine the report and feedback to the health facility for any follow up visits to the patients. A 46-year old pharmacist shared his views this way on the issue:

*“Yes there is a procedure and what usually happens is that there is an institutional contact person that is the pharmacist, who is supposed to compile all the side effects people come to complain about. There is a form that you are to record all these complaints which will be forwarded to Food and Drug Authority (FDA) at the national level for their study and advice. After that the report is sent back to the institution for their study and action based on the advice offered by FDA. There are various possibilities that can cause a person to react to medication. It could be wrong usage, the human system, combination of different drugs at the same time among others. So we have to monitor the person and make sure that he/she takes the drug correctly and if the same side effects are presented then you have to change treatment for the person and see”* (IDI: 46-year old pharmacist).

### Factors affecting adverse events reporting

Provider and patient-related factors were the two broad factors identified to be responsible for the low reports of adverse drug reactions in the study area.

### Provider-related factors

The providers reported that, doctors, pharmacists and dispensary technicians who are required to educate patients on adverse drug events are few and the pressure of work leaves them no time to discharge such duties. The absence of qualified human resource to serve the high number of patients has made some of the facilities to settle for less qualified technicians who do not have the capacity to provide such education. Also, at the community level, community members are not routinely told during sensitization programmes or at the dispensary level to report adverse events.

*‘That is the issue, usually, they are supposed to be told about it either at the OPD level or at the dispensary when they are issued with the medications. The problem is that it doesn’t normally happen because we do not even have qualified staff at the dispensary to do the education. The language is also a problem and I remember when I was at Sandema, because of the language barrier, I could not communicate with the patients. Quite apart from that we do not have enough staff at the dispensary and that is also a factor because the few people who are there are usually under pressure to serve patients and as a result, there is no time to be able to educate patients on all these issues* (IDI: 42-year old Pharmacist).’

Lack of awareness of their responsibility when adverse events occur was the main reason for not reporting to health facilities according to the patients who were interviewed.

*“Many of them (refers to health workers) do not give that information to us. Instead of them to tell you that when you take the drug and get side effects, you should come back to complain they don’t do that. They will just give you the drugs and ask you to go and take and that is all. If they were to tell you that when you take the drug you will get this or that problem and when it happens like that you should come back to the hospital, they don’t do that”* (IDI: 38-year old male patient).

*“Because they have not asked me to come back; let’s say if the doctor gives you the drug and tells you to come back when you get some disturbances after you have taken the drug then you have to go back but if the doctor does not tell you to come back you will not go. Actually, they have never given me a drug and ask me to come back if I take the drug and get some disturbances with the drug”* (IDI: 42-year old female patient).

The patients attributed the low reporting to the unprofessional conduct of some health workers which deterred patients from returning to report adverse events. Health professionals were reported to be verbally abusive and patients did not find it pleasant to have encounters with them unless it was absolutely necessary. A female patient had this to say:

*“Yeah at times when you go back to the health facility, they would shout at you and say that is the way the drug works; that is the way the drug works and if you don’t want them to shout at you then you will not go back and tell them and rather prefer to keep it. So when you go for the first time and they shout at you that way, when you take it again and it happens like that you will not go?”* (IDI: 31-year old female patient).

Fear of abuse also prevented patients who sought treatment outside health facilities from reporting adverse events. The participants reported that this was common with patients who suffered side effects due to non-adherence to the dosing regimen because they were likely to be verbally abused by the health workers. This is how a female patient expressed her concerns.

*“The reason is that some people usually do not have money to go to the hospital and would usually go and buy cheap drugs at the drugstore. The problem is that when they take the drugs wrongly and get problems (side effects) at home, they are afraid to go and tell the doctor because they do not know what the doctor would say and sometime, they could be insulted by these health workers. This is the one other reason why such people who get these side effects do not report”*(IDI: 26-year old female patient).

### Patient-related factors

Patients are usually not motivated to come back and report adverse events especially if the side effects are those already known to be associated with the drug. Patients perceived such side effects as normal and reporting them held no relevance to them. Patients preferred to endure such side effects at home rather than report them to the health facility with the reason that every drug has a side effect and therefore it was normal to experience them.

*“The reason is that I know the way it works and that made me not to go back and I was waiting to see what would happen after the third day….So because I know how the drug works is the reason why I did not go back to tell them”* (IDI: 30-year old female patient).

Participants also identified long queues at the OPD that led to long waiting-time before receiving care as a critical factor that affect the reporting of adverse events. To them, such experiences were not worth repeating just to report an adverse event. Patients preferred to spend time on their farms than to traveling to health facilities to report adverse events.

*“It is because of the long queues at the health facilities where we usually have to go and spend the whole day before seeing the doctor makes it difficult for us to go back when we get some of these problems after using the drugs*” (IDI: 29-year old female patient).

*“…you know most of the women here are business women and some of them are farmers,. So if they have the side*-*effects and they can still walk they will prefer to go to their farms and work rather than coming to the facility. And others will say that if I am strong enough to go to the facility why don’t I just go to the market and manage my business, so mostly that is the reason why they don’t come”* (IDI: 29-year old health worker).

Other patients said that previous reports of side effects yielded no benefits to them. They were therefore not motivated to report subsequent events because they did not see the relevance of the exercise. Peers who heard their stories were not also motivated to report their own experience with ACT to the facilities.

*“You know when I went and complained to them they did not do anything about it, they rather said it is the drug and it would go. Now that they said it is as a result of the drug and nothing else why then would you waste your time going if you take the drug again and get these side effects. When I talk about it for other people to know, they will not also go back to the health facility when they take the drug and experience those things because they know it is the drug that is working”* (IDI: 44-year old female patents).

### Suggestions on how to improve adverse events reporting

Various suggestions were given by participants to help improve on adverse events reporting. Most of the patients were of the view that health education should be given to patients in order to encourage them to report these side effects.

*“…drug store attendants and the health workers at the hospitals should often inform patients of the possible side effects and also tell them to come back if they experience serious side effects after taking the drugs so that they can test you and see what the problem is*”(IDI: 38-year old female patient).

The health workers said that most people were ignorance and at such did not know that they were supposed to come back and report and if patients were reminded continuously each time they were given ACT, it would have a positive impact on adverse event reporting in the study area.

*“…we (health workers) should have patience towards our clients and try to educate them about the side effects and also encourage them to report when they get these side effects at home….”* (IDI: 41-year old health worker).

Most of the patients said that it was important for health workers to have patience and behave nicely toward their clients in a manner that would encourage them to always feel free to visit the health facility anytime they have health problems. Patients were of the opinion that there should be a policy whereby people who took ACT and got side effects should not be made to join long queues at the hospital during review visits.

*“The nurses should have patience when dealing with people at the health facility. They should tell the people to feel free and come back to the health facility anything they take these drugs and get side effects….”* (IDI: 25-year old female patient).

*“…The doctors should make it such that when you take the drugs and get side effects you should walk straight to the doctor and complain to him, it should not look like you are coming for the first time where you would go and follow line for long before you see the doctor to give your complain about the side effects you got from using the drugs they gave you*” (IDI: 39-year old female patient).

### Limitations

Though, this study provides information on factors affecting adverse events reporting of ACT, some limitation however must be considered. Most of the interviews were conducted in the main local languages in the study area (Kasem and Nankani) and some people were engaged to transcribe and translate the recordings into English. It is possible that certain individuals could introduce some level of bias in few of the statements made in the local languages in trying to translate them into English, which could have affected the original meaning. To help alleviate this, the transcripts were edited by the study team in order to make sure that the transcriptions and translations were properly done.

## Discussion

The study presents reported side effects as are experienced from using ACT to treat uncomplicated malaria using qualitative approach. Body weakness and dizziness are the most frequent side effects many of the patients reported. Majority of the patients who took artesunate-amodiaquine and artemether-lumefantrine, respectively, reported to have experienced side effects such as body weakness, dizziness, itching and abdominal pain [5–14]. In this study however, views expressed are slightly different because most patients attributed all the side effects to the use of artesunate-amodiaquine compared to other combinations.

According to Asante et al., side effects such as headache, inability for some patients to control the neck and tongue were reported by patients who used ACT [5]. These side effects have not been reported in this study. However, swollen testes was reported in this study as one of the side effects of ACT. It is possible that swollen testes may not be as a result of the use of ACT as reported by participants. It could be that this coincided with the use of the medication by the person and because of that it was attributed to it. It is possible that these differences in views on the side effects on the use of ACT could be as a result of differences in individual human system.

Patients are supposed to eat very well before using these medications. According to the study participants, patients’ inability to eat food before using the drugs made them to experience these side effects. When the artesunate-amodiaquine was first introduced in Ghana, many patients who used it had several side effects because the components of the drug were very high [7]. Opinions from patients who use artesunate-amodiaquine in this study suggested that the components are still high and that could be the reason why they are still experiencing these side effects. It is demonstrated that poor noncompliance to guidelines to the use of medications accounted for drug side effects [15]. The health professionals explained that some malaria patients only initiate treatment when the condition is severe and that this was responsible for some of the side effects and not necessarily the ACT. They added that poly-pharmacy was common within the health system and that drug interaction was more likely to cause some of the side effects that the patients complain about. The health workers reported non-adherence to the dosing regimen as a significant contributor to side effects. They were particularly concerned about overdosing by some patients.

In Ghana, the policy is that all patients are supposed to be well informed by prescribers and pharmacists on the possible side effect they are likely to experience from the use of ACT and the need for them to report. However, people are not being encouraged to report when they experience these side effects. This study solicited views from patients on the factors affecting adverse events reporting. Two broad factors were identified (health facility factors and patient factors) to be responsible for low reporting of adverse events. Earlier study reported that because of lack of knowledge, seventy-five percent (75) % of those who experienced side effects did not report to health workers [4].

Based on the views shared by participants, health workers are not educating patients on the possible side effects they are likely to experience and the need to report them. The inability of health professionals to give the necessary health information to patients directly affects adverse events reporting [4]. The problem could be resolved if efforts are made by health workers who prescribe ACT to patients to provide the needed health educate on these drugs to them. Long waiting time at health facilities and negative health worker attitude discouraged patients from attending health facilities for health care [15, 16]. These were also mentioned as factors affecting effective side effects reporting in this study.

The fact that patients who previously reported side effects at the health facility and could not get any help from health workers has been reported to have negative effect on adverse events reporting. Patients are expected to be taken care of by health providers when they visit the health facility for medical care. It is, therefore, normal that when patients are not well looked after, they would not be encouraged to visit the health facility when they have similar health issues in future.

Generally, reports on adverse effects of drugs were rare within the health facilities. A few facilities, mostly the health centres, reported completing the adverse drug reporting form for a few cases. These facilities reported using feedback from the FDA, to observe patients who suffered particular side effects during subsequent treatments, Most of the community health compounds had never documented any side effect and they did not have the appropriate forms to capture adverse events.

## Conclusion

The key factors responsible for the inability of patients to report side effects from using ACT are lack of health education, attitude of some patients and the unpleasant behaviour of some health workers towards patients at the health facilities. Despite the fact that some level of health education has been given to patients by health workers on the use of ACT and how to prevent malaria, there is a need for health workers to specifically educate patients on the side effects of ACT.

It is, therefore, recommended that intensive and continued education should be incorporated into the routine health care activities. Patients who have been given ACT should be informed and encouraged by health workers, pharmacists and chemical shop owners to quickly report any adverse events they would have experienced from using ACT. Also, patients who experience side effects should be properly taken care of at the health facility during review visits. If possible, a different consultation room should be created only for such purpose at the health facilities. This will encourage patients who experience side effects to report them.
